# Effect of changes in tidal volume on multiple breath washout outcomes

**DOI:** 10.1371/journal.pone.0219309

**Published:** 2019-07-03

**Authors:** Felix Ratjen, Renee Jensen, Michelle Klingel, Reginald McDonald, Courtney Moore, Nick Benseler, David Wilson, Sanja Stanojevic

**Affiliations:** 1 Translational Medicine Research Program, The Hospital for Sick Children, Toronto, Canada; 2 Division of Respiratory Medicine, The Hospital for Sick Children, Toronto, Canada; 3 University of Toronto, Toronto, Canada; UKBB Universitats-Kinderspital, SWITZERLAND

## Abstract

The lung clearance index (LCI), measured by multiple breath washout (MBW), reflects global ventilation inhomogeneity and is a sensitive marker of early obstructive airway disease. For the MBW test to accurately reflect a subject’s gas mixing within the lungs, the breathing pattern should represent physiologically appropriate tidal volumes (V_T_) and respiratory rate (RR). We aimed to assess whether changes in V_T_ impact MBW outcome measures with a series of prospective and retrospective studies. MBW testing was performed using the Exhalyzer ^®^ D (EcoMedics AG, Switzerland). Healthy adult subjects performed MBW with uninstructed tidal breathing and a series of instructed tidal breathing tests, designed to isolate specific features of the breathing pattern. In addition, we retrospectively analyzed MBW data from two pediatric multi-centre interventional studies of cystic fibrosis (CF) subjects to determine the range of V_T_ observed during uninstructed breathing, and whether breathing outside this range impacted results. The LCI was lower, but not significantly different between deep breathing at 20 ml/kg body weight and uninstructed tidal breathing; whereas LCI was significantly higher during shallow breathing compared with normal tidal breathing. For the majority of subjects with CF (80%), V_T_ ranged from 9-15mL/kg. Within the observed V_T_ range, LCI was similar in trials with mean V_T /_kg below this range compared to trials with V_T_ /kg within the range. If subjects breathe naturally and are not instructed to use specific targets, the range of V_T_ is within physiologically appropriate limits and normal variations observed do not impact MBW outcomes.

## Introduction

The lung clearance index (LCI), measured by multiple breath washout (MBW), reflects global ventilation inhomogeneity and is a sensitive marker of early obstructive airway disease.[1–3] The MBW test does not require specific breathing maneuvers, thus there is growing interest in the use of MBW to monitor disease progression and treatment efficacy in children with lung disease.[4]

For the MBW test to accurately reflect a subject’s gas mixing within the lungs, the breathing pattern should be representative of physiologically appropriate tidal volumes (V_T_) and respiratory rate (RR). The 2013 European Respiratory Society/American Thoracic Society consensus statement for the MBW test provided general recommendations on test procedure but only broadly outline quality criteria for normal tidal breathing pattern.[5] A V_T_ range of 10–15 ml/kg, based on measured weight, has been adopted as the default breathing range for the Exhalyzer D, a commonly used nitrogen multiple breath washout (N_2_MBW) device. However, this range may not be representative of subjects’ ‘normal’ V_T_ and may force subjects to breathe outside their physiological range. Targets based on measured weight may disproportionally influence under-weight or over-weight subjects. This issue can be resolved using a standard fixed V_T_ target,[6] but the recommended 1L V_T_ target has been shown to overestimate ventilation inhomogeneity and significantly changes functional residual capacity (FRC) in children.[7]

In this study we aimed to systematically assess whether changes in V_T_ impact N_2_MBW outcomes, with a series of prospective and retrospective analyses: 1) increasing and decreasing V_T_ outside the target range in healthy subjects, 2) applying weight based V_T_ targets in under-and overweight children, and 3) describing the range of V_T_ observed during uninstructed breathing and investigating whether breathing outside this range affects outcomes.

## Methods

### N_2_MBW measurement

N_2_MBW testing was performed using the Exhalyzer D (EcoMedics AG, Switzerland). Operators were trained and certified in data collection according to current consensus guidelines.[5, 8] All N_2_MBW traces were reviewed for technical acceptability by centrally trained reviewers using a standardized protocol.[5, 9] All subjects or parents/guardians for children under the age of 18 years provided written consent (children provided written assent where appropriate) and research ethics approval was obtained from the Hospital for Sick Children Research Ethics Board (REB#1000051399)

### Altering V_T_ in healthy adults

To systematically determine the effect of varying V_T_ on N_2_MBW outcomes, healthy adult subjects performed a N_2_MBW test with uninstructed tidal breathing (first test) and a series of instructed tidal breathing tests in random order, designed to isolate specific features of the breathing pattern. Subjects were instructed to use visual V_T_ targets to breathe with 1) shallow breaths at 5ml/kg of measured body weight (5ml/kg below the current software recommended lower limit) and 2) deep breaths of 20ml/kg (5ml/kg above the current software recommended upper limit). Subjects then performed two additional tests, in random order, where they were instructed to breathe at 3) a RR increased by 5 breaths per minute, and at 4) a RR decreased by 5 breaths per minute compared to their normal RR. Respiratory rate (RR) was controlled by a metronome and calculated relative to the subject’s uninstructed V_T_ to maintain normal minute ventilation (within +/- 25% of the uninstructed trials). The differences in outcomes between conditions were analyzed using repeated measures ANOVA analysis, followed by pairwise comparisons using a Bonferroni correction.

### Effect of V_T_/kg targets in under-and over-weight children?

Weight based tidal volume targets (V_T_/kg) are often used to guide breathing pattern during MBW testing but may encourage physiologically inappropriate tidal volumes (too small or too large) in subjects who are under or overweight. To demonstrate the effect of using breathing targets inappropriately derived from measured weight, we measured underweight and overweight subjects,[10] free of respiratory disease recruited from sub-specialty clinics at the Hospital for Sick Children. Each subject performed two N_2_MBW tests: 1) with V_T_ targets (8 to 10 mL/kg) defined using measured weight (kg_-M_) and 2) with V_T_ targets (8 to 10 mL/kg) defined using ideal body weight (IBW; kg_-IBW_), where IBW was calculated as the 50^th^ weight-for-age centile [11] for girls less than 14 years of age, and boys less than 18 years of age, and sex-specific IBW values for post-pubertal subjects.[12, 13] Subjects were coached to ensure target V_T_ ranges were maintained. A target of 8 to 10 mL/kg was chosen to reflect uninstructed V_T_ observed during the adult measurements.

### Effect of V_T_ variability on N_2_MBW outcomes during uninstructed breathing

To define the range of V_T_ observed when subjects were not instructed to breathe to a specific target, we retrospectively analyzed N_2_MBW data from two pediatric (2.5–11 years old) multi-centre interventional studies of subjects with cystic fibrosis (CF), blinded to the intervention allocation.[14, 15] Subjects were intentionally distracted (e.g. TV show), but no additional coaching or visual incentives (e.g. Spiroware software V_T_ targets) were used. Trials with leak or those that did not reach end of test were **excluded** from analysis; trials with irregular breathing pattern were **included** in analysis. Average V_T_ was calculated as the mean expiratory volume of all washout breaths. The normal/unrestricted V_T_ range was defined as the 10^th^ to 90^th^ percentile of tidal volume measured during uninstructed breathing. We summarized the frequency and magnitude of deviations above and below the observed V_T_ range both within trials and within-subjects on the same test occasion. To determine whether observed deviations outside the observed V_T_ range affect MBW outcomes, we compared LCI, cumulative expired volume (CEV) and FRC from trials to the average V_T_ within the same subject and same test.

### Effect of sighs on N_2_MBW outcomes

A sigh was defined as any washout breath with an V_T_ greater than 1.5 times the mean V_T_ of the trial. To determine if sighs affect N_2_MBW outcomes, we compared outcomes between trials with one or more sighs to the average of all the other acceptable trials in the same test occasion. Sighs during the pre-phase, the first breath of the washout or that resulted in increased N_2_ concentration related to trapped gas release were not included in analyses.

## Results

### Altering V_T_ in healthy adults

Twelve healthy adult subjects (mean age 23.5 years (SD 5.9)), with normal BMI (mean 23.0 kg/m^2^ (SD 3.5)) performed N_2_MBW under a series of instructed tests. During uninstructed breathing, the mean (SD) tidal volume was 8.9 (1.9) ml/kg. LCI was lower, but not significantly different between deep breathing at 20 ml/kg body weight and uninstructed tidal breathing (Table 1); whereas LCI was significantly higher during shallow breathing compared with normal tidal breathing (Table 1). The effect of shallow breathing on LCI was driven by larger changes to the CEV, relative to changes in the FRC (Table 1). During deep breathing there were proportionate changes in both the CEV and FRC. Compared to normal breathing, shallow breathing resulted in a significantly higher dead space volume (V_D_)/V_T_ ratio, whereas deep breathing resulted in a significantly decreased V_D_/V_T_. During shallow breathing there was a negative correlation between V_D_/V_T_ and LCI (r = -0.73, 95% CI -0.94, -0.30; p = 0.01); no correlation was seen during deep breathing. Shallow breathing did not affect the V_T_/FRC, whereas V_T_/FRC was significant higher during deep breathing (Table 1).

**Table 1:** Changes in MBW outcomes in healthy adults at different tidal volumes (V_T_).

Lung function measure	Shallow Mean(SD)	Uninstructed Mean(SD)	Deep Mean(SD)	Shallow vs Uninstructed Mean difference (95% CI)	Deep vs Uninstructed Mean difference (95% CI)
**LCI**	8.87 (1.39)	7.70 (0.82)	7.12 (0.59)	1.16 (95% CI 0.31, 2.02) p = 0.01	-0.58 (95% CI -1.44, 0.27) p = 0.17
**FRC (L)**	2.82 (0.92)	3.02 (0.90)	2.80 (0.94)	-0.22 (95% CI -0.41, -0.02) p = 0.03	-0.20 (95% CI -0.40, -0.01) p = 0.04
**V_T_(mL)**	290 (50)	538 (137)	1304 (256)	-247 (95% CI -364, -131) p<0.001	766 (95% CI 650, 883) p<0.001
**CEV (L)**	25.5 (10.3)	23.7 (9.4)	19.7 (6.0)	1.8 (95% CI -1.5, 5.1) p = 0.29	-4.0 (95% CI -7.4, -0.7) p = 0.02
**V_D_/V_T_**	0.238 (0.021)	0.219 (0.039)	0.123 (0.029)	0.037 (95% CI 0.020, 0.054) p<0.001	-0.096 (95% CI -0.127, -0.065) p<0.001
**V_T_/FRC**	0.112 (0.037)	0.189 (0.070)	0.517 (0.200)	-0.078 (95% CI -0.164, 0.008) p = 0.08	0.328 (95% CI 0.241, 0.414) p<0.001

MBW outcomes assessed include the lung clearance index (LCI), functional residual capacity (FRC), tidal volume (V_T_), cumulative expiratory volume (CEV) and dead space volume (V_D_). SD: Standard Deviation, CI: Confidence Interval

Insignificant changes in end-tidal CO_2_ of 0.21% (95% CI -0.22, 0.64) and -0.64% (95% CI -1.07, -0.21) were observed during shallow and deep breathing, respectively. None of the N_2_MBW outcomes were affected by breathing at faster and slower respiratory rates compared to uninstructed breathing (Table 2).

**Table 2:** Changes in MBW outcomes in healthy adults with differing respiratory rates (RR).

MBW outcome	Lowest RR Mean(SD)	Uninstructed RR Mean(SD)	Highest RR Mean(SD)	Lowest vs Uninstructed Mean difference (95% CI)	Highest vs Uninstructed Mean difference (95% CI)
**LCI**	7.17 (0.49)	7.30 (0.47)	7.33 (0.58)	-0.12 (95% CI -0.29, 0.05) p = 0.14	0.03 (95% CI -0.15, 0.21) p = 0.70
**FRC (L)**	2.97 (1.07)	2.99 (1.04)	3.02 (1.04)	-0.02 (95% CI -0.19, 0.15) p = 0.83	0.03 (95% CI -0.10, 0.16) p = 0.60
**V_T_ (mL)**	608.9 (124.3)	601.9 (139.4)	593.3 (139.6)	7.0 (95% CI -7.2, 21.2) p = 0.30	-8.6 (95% CI -21.1, 3.9) p = 0.16
**CEV (L)**	23.7 (8.8)	24.2 (8.7)	24.4 (8.6)	-0.6 (95% CI -2.2, 1.1) p = 0.46	0.2 (95% CI 0.9, 1.2) p = 0.71

MBW outcomes assessed include the lung clearance index (LCI), functional residual capacity (FRC), tidal volume (V_T_) and cumulative expiratory volume (CEV). SD: Standard Deviation, CI: Confidence Interval

### Effect of V_T_/kg targets in under- and overweight children

As proof of principle, three subjects with extreme weights (BMI centiles 11.2^nd^, 99.1^th^ and 99.8^th^) completed two N_2_MBW tests where the target V_T_ was defined using 1) measured weight, and 2) ideal body weight defined as the 50^th^ weight-for-age centile.[11] For the underweight subject, V_T_ targets based on measured weight resulted in an increased LCI compared with ideal weight V_T_ targets. In the two overweight children, V_T_ targets based on measured weight resulted in lower LCI values compared with V_T_ targets based on ideal weight. The heaviest subject, had a 12% increase in LCI when instructed to breathe using V_T_ outside a physiologically appropriate range (Table 3).

**Table 3:** Characteristics of subjects at both ends of the weight spectrum under the condition of measured weight, and ideal weight.

	BMI kg/m^2^ (centile)	Weight entered	Weight (kg)	V_T_ Target Range	LCI (CV%)	FRC (L) (CV%)	CEV (L)	V_T_ (mL)	mL/kg of IBW	No. of breaths	V_T_ mean/FRC	Minute Ventilation (l/min)
**1**	16.8 (11.2)	Measured	52.5	420–525	6.54 (0.6)	3.07 (0.6)	22.6	493	7.63	46	0.161	10.3
		Ideal	64.6	517–646	6.13 (1.3)	3.22 (0.3)	21.6	644	10.0	33	0.200	10.7
**2**	30.5 (99.1)	Measured	70.4	563–704	8.24 (3.8)	1.34 (1.3)	12.0	737	17.7	16	0.549	7.56
		Ideal	37.2	298–372	8.57 (2.2)	1.25 (1.6)	12.3	420	10.1	29	0.337	9.00
**3**	52.0 (99.8)	Measured	143.0	1144–1430	8.38 (2.1)	1.99 (6.7)	17.4	1327	25.0	13	0.669	12.0
		Ideal	44.2	354–442	9.44 (4.1)	1.88 (5.7)	19.5	598	11.3	33	0.317	9.62

MBW outcomes assessed include the lung clearance index (LCI), functional residual capacity (FRC), cumulative expiratory volume (CEV) and tidal volume (V_T_).

### Effect of V_T_ pattern during uninstructed breathing on N_2_MBW outcomes

**Fig 1:**
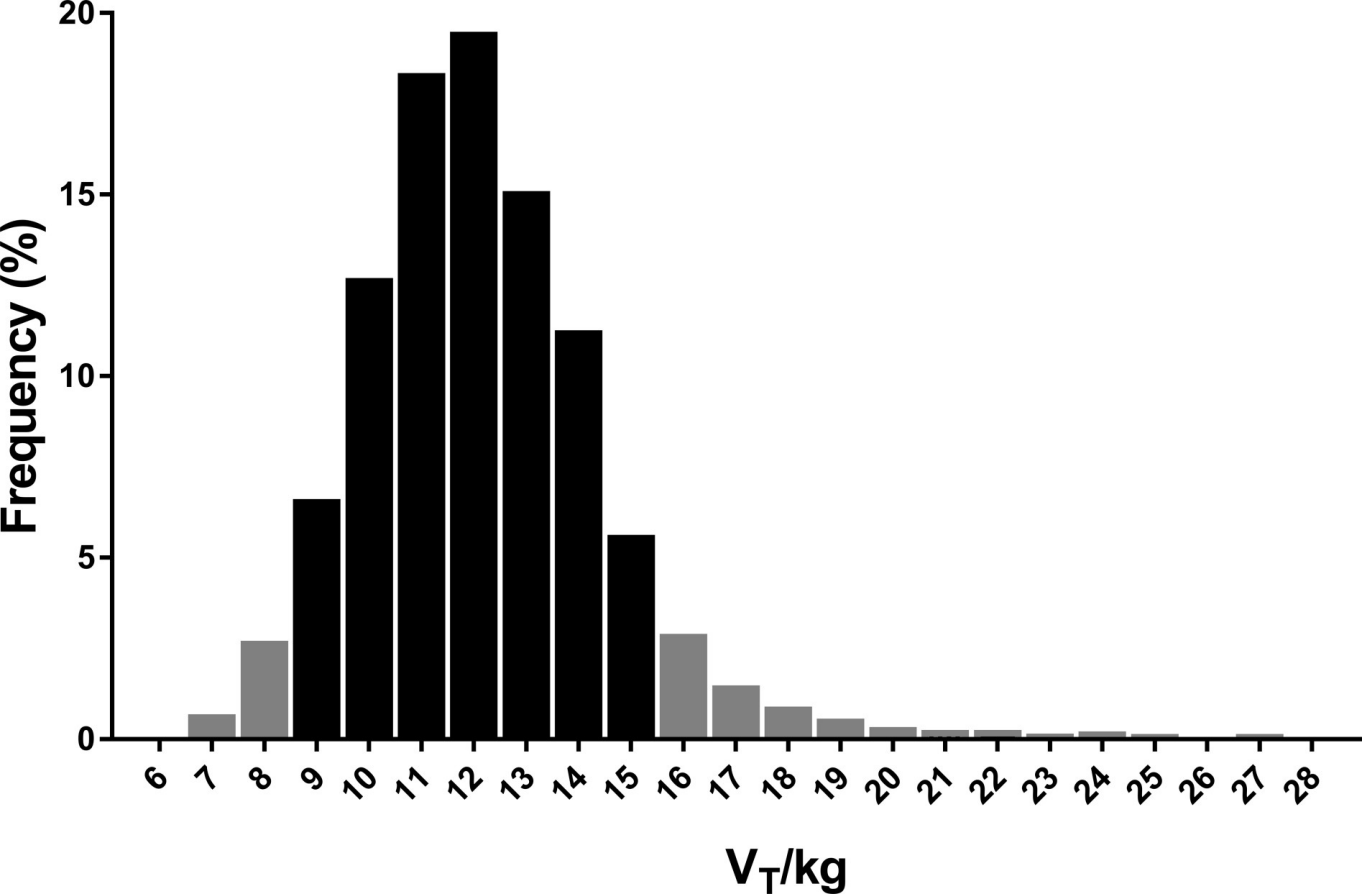
Distribution of tidal volumes (V_T_) observed in subjects with cystic fibrosis. Eighty percent of subjects had a V_T_ within 9–15 ml/kg_IBW (ideal body weight)_. There were 9 trials with a V_T_/kg > 28mL/kg but are too few to be visible.

The average V_T_ range from 7091 trials from 428 subjects (57% female, median age (range) 7.9 years (2.2, 12.9)) from two multi-centered interventional studies was 12.2 mL/kg (SD 2.6) ranging from 4.7 to 38.1 mL/kg. The observed range of V_T_ (10^th^– 90^th^ percentile) fell between 9-15mL/kg (Fig 1) in 80% of all trials.

**Fig 2:**
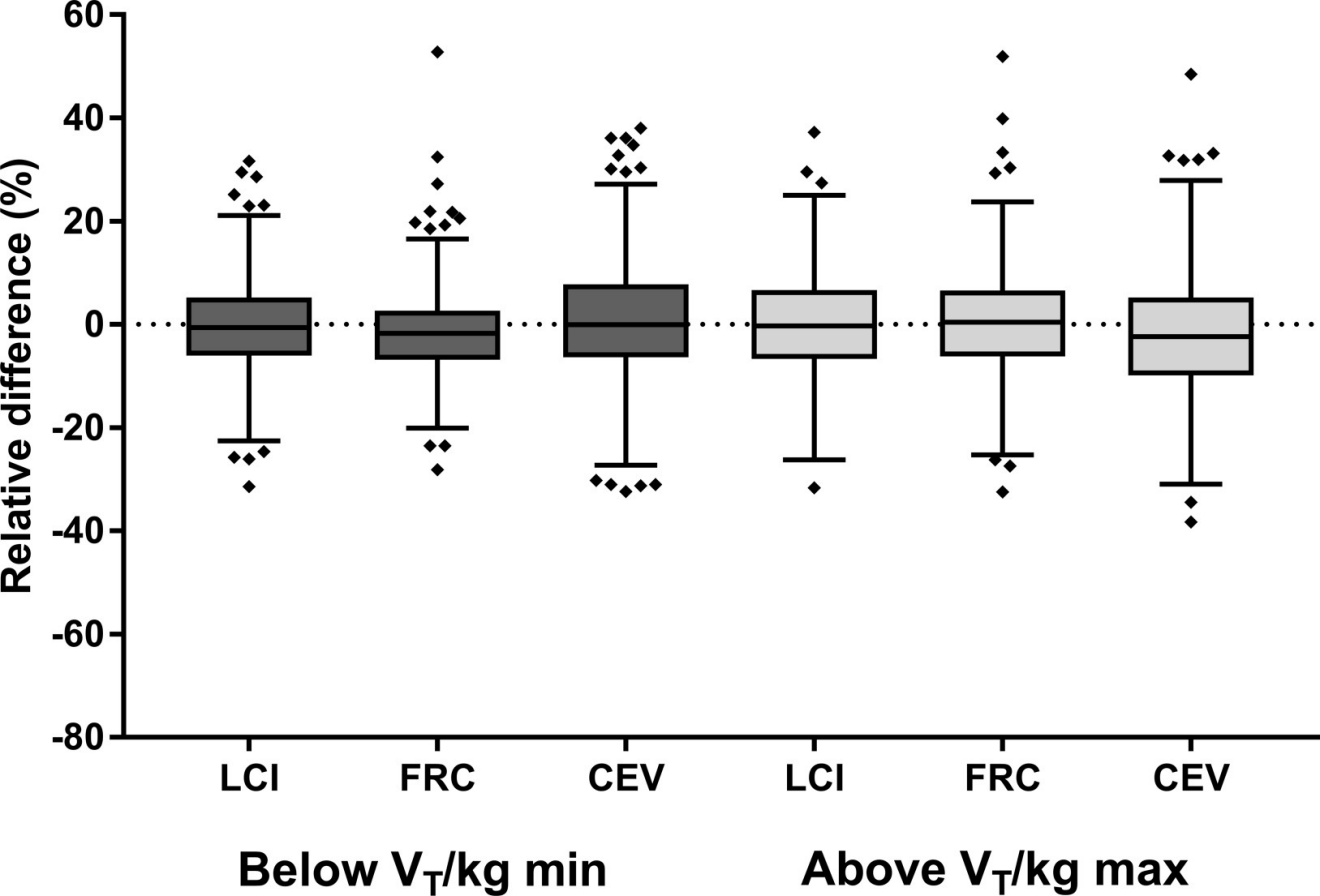
Relative difference in MBW outcomes for trials with V_T_ below 9 ml/kg (black boxes) and those above 15 ml/kg (grey boxes) compared to same-visit trials that fell within the ideal tidal volume (V_T_) range. MBW outcomes assessed include the lung clearance index (LCI), functional residual capacity (FRC) and cumulative expiratory volume (CEV).

The number of trials where the average V_T_ for the entire trial was < 9 ml/kg was small (437/7097 or 6.2%), as was the absolute magnitude of the difference in V_T_ (median difference 0.6 ml/kg; IQR 0.2–1.1 ml/kg). There were no trials with a mean Vt/kg less than 5 ml/kg observed. A greater number of trials above the ideal V_T_ range were observed (714/7097 or 10.0%), but the magnitude of the difference was minimal (1.3ml/kg (IQR 0.5–3.3ml/kg)). Only 109/7097 (1.5%) trials had breaths that were greater than 20 ml/kg_._ Within the observed range of V_T_, LCI was similar in trials with mean V_T/_kg below the normal tidal volume range compared to trials with V_T_/kg within the normal range (Fig 2), as was the LCI for trials with mean V_T/_kg above the tidal volume range (mean LCI difference 0.04 units (95% Confidence Interval -0.14, 0.05) (Table 2).

A similar pattern was observed within a trial; the vast majority of breaths fell within the ideal V_T_ range, and it was more common for subjects to breathe with slightly higher volumes (15–20 ml/kg) than smaller volumes (5–9 ml/kg). The V_T_ variability within a test (coefficient of variation (CV) of V_T_ across all breaths) was not correlated with LCI (r = -0.153), nor the CV of LCI within trials from the same test occasion (r = 0.127).

### Effect of sighs on MBW outcomes

Sighing was a relatively infrequent event; 60% of trials did not have a sigh. When a sigh did occur within a trial, it was most often a single event (57%). On average LCI was reduced by 0.05 LCI units (0.9%) when there was a sigh within a trial, compared to trials in the same subject without a sigh (Table 4).

**Table 4:** Effect of breathing outside the ideal V_T_ range in children with CF.

	<9ml/kg (n = 246)	>15ml/kg (n = 409)	Sighs (n = 1856)
	Absolute Difference from ideal (95% Confidence Interval)		
LCI	-0.05 (-0.18, 0.07)	-0.04 (-0.14, 0.05)	-0.05 (-0.09, -0.01)
FRC (L)	-0.02 (-0.03, -0.00)	-0.01 (-0.02, 0.00)	-0.02 (-0.03, -0.02)
CEV (L)	0.14 (-0.10, 0.38)	-0.33 (-0.47, -0.19)	-0.24 (-0.30, -0.18)

## Discussion

Tidal volumes in children and adults performing the N_2_MBW test range from 9-15ml/kg, and generally do not exceed these limits. Although very shallow breathing results in higher LCI values, most subjects do not naturally breathe at these extreme ranges. In normal weight individuals, target V_T_ ranges based on measured weight provide a reasonable range compatible with uninstructed breathing, whereas physiologically inappropriate V_T_ targets in under–and–overweight individuals can lead to erroneous outcome measures. Using ideal weight targets based on the average weight observed in healthy children (e.g. 50^th^ centile weight-for-age) can provide a reasonable V_T_ target to avoid forcing subjects to breathe outside a physiologically appropriate range. Variations in V_T_, including sighs that do not result in increases in the end-tidal nitrogen concentration, do not affect N_2_MBW outcomes and should not be used to exclude trials from reported values. Although uncommon during uninstructed breathing, extreme variations in V_T_ can affect N_2_MBW outcomes, and therefore operators should monitor breathing and ensure the subject is breathing comfortably.

The impact of small and large tidal volumes on MBW outcomes in the adult subjects demonstrates the importance of allowing subjects to breathe within reasonable V_T_ limits. LCI was significantly higher during shallow breathing (5 ml/kg), which is in agreement with a previous report assessing the influence of both changing body position and increasing tidal volume in fixed intervals over the range reflected in the current study.[16] Unlike Yammine et al., we did not see changes in LCI at higher tidal volumes, which is likely because our limits were much lower than the equivalent at 1L fixed volume.[7] As LCI is calculated as the ratio of CEV and FRC (LCI = CEV/FRC) we investigated the impact of tidal volume on each of these. The increase in CEV observed when subjects breathe at small tidal volumes, is likely a result of the increased dead space to tidal volume ratio (V_D_/V_T_).[17] Interestingly, FRC decreased in a majority of subjects during shallow breathing, rather than increasing as expected. This may be due to the method of FRC calculation (FRC = FRCgas sampling−pre-gas sampling point V_D_); low tidal volumes alter the V_D_/V_T_ ratio, which has an impact on the FRC measurement.[17] In infants, Schmalisch et al. observed a strong correlation between LCI, V_D_/V_T_ and V_T_/FRC,[18] a finding replicated by Benseler et al. in adults when equipment dead space volumes were systematically increased.[17] When the V_T_ is artificially increased outside a normal physiological range, the V_D_/V_T_ ratio decreases while the V_T_/FRC ratio increases, resulting in a lower LCI. This was more frequently observed in taller subjects with BMI at the lower range of normal. Both CEV and FRC changed with increased V_T_, which is important if these results are reported and interpreted independently, even if the resulting changes in LCI are minimal.

### Limitations

All N_2_MBW testing was performed exclusively on the Exhalyzer D device, and may not reflect the equipment dead space and resistance of other devices, which may influence breathing patterns. Additionally, the effect of V_T_ on MBW outcomes measured with other inert tracer gases was not assessed. The observed V_T_ ranges and associations with N_2_MBW outcomes were made in healthy adult subjects and children with CF who were free of respiratory symptoms at the time of test, and may not be generalizable. In CF subjects with more advanced disease, where larger lung volumes may recruit under-ventilated regions of the lung, LCI may be more influenced at larger tidal volumes.[7] Breathing within the recommended V_T_ range will likely reduce this effect. We only evaluated V_T_, and breathing pattern *per se* was not investigated. All N_2_MBW data were collected from trained and certified operators, and may not be generalizable to naïve operators. Training operators to be able to recognize and encourage a natural and physiologically appropriate V_T_ and RR will help to ensure that N_2_MBW outcomes are not biased by extreme tidal volumes. Finally, we only compared trials within a single test occasion and are not able to comment on how variability in tidal volume between test occasions affects interpretation of repeated measurements.

In conclusion, when subjects breathe naturally and are not instructed to use specific targets, the range of V_T_ is within physiologically appropriate limits and normal variations observed do not impact N_2_MBW outcomes. Subjects forced to breathe outside of physiologically appropriate limits may alter N_2_MBW outcomes and lead to incorrect interpretation.
